# New Ultrasound-Guided Approach to Access to the Posterolateral Part of Intervertebral Lumbar Discs: A Cadaveric Study

**DOI:** 10.3390/jcm13154411

**Published:** 2024-07-28

**Authors:** Jacobo Rodríguez-Sanz, Sergio Borrella-Andrés, Carlos López-de-Celis, Isabel Albarova-Corral, Albert Pérez-Bellmunt, Elena Bueno-Gracia, Miguel Malo-Urriés

**Affiliations:** 1 Faculty of Medicine and Health Science of Universitat Internacional de Catalunya, 08195 Sant Cugat del Vallés, Spain; jrodriguezs@uic.es (J.R.-S.); aperez@uic.cat (A.P.-B.); 2 ACTIUM Functional Anatomy Group, 08195 Sant Cugat del Vallès, Spain; 3 Health Sciences Faculty, Department of Physiatry and Nursing, University of Zaragoza, 50009 Zaragoza, Spain; sergiocai04@gmail.com (S.B.-A.); ialbarova@unizar.es (I.A.-C.); ebueno@unizar.es (E.B.-G.); malom@unizar.es (M.M.-U.); 4 Fundació Institut, Universitari per a la Recerca a l’Atenció, Primària de Salut Jordi Gol i Gurina (IDIAPJGol), 08028 Barcelona, Spain

**Keywords:** ultrasound, intervertebral lumbar disc, invasive, cadaver

## Abstract

**Background:** Approximately 40% of chronic low back pain patients have a discogenic origin. In relation to intervertebral disc injuries, most of them are in the posterior and lateral zone of the disc, involving the anterior lumbar roots and the spinal cord. **Objective**: The objective was to analyze and describe the accuracy and safety of a new ultrasound-guided approach to target the posterolateral part of the intervertebral lumbar discs in cadaveric specimens. **Methods**: A cross-anatomical study on sixty cadaver intervertebral lumbar discs was performed. A needle was introduced in the posterolateral part of the discs using ultrasound guidance. A transducer was placed in the anterior abdomen to visualize the discs in cross-section as well. A dissection of the specimen was performed to visualize the final position of the needle tip and its distance from the main lumbar structures. The angulation, length, and distance of the needle from the vertebral spine, the relevant ultrasound anatomical references, and the accuracy of the procedure were evaluated. **Results**: The needle tip reached the posterolateral part of the discs in 93.3% of the attempts. The mean length of the needle inserted was 79 ± 15 mm, the angulation 129 ± 20.2°, the distance from the spinous process was 77 ± 19 mm, and the distance of the needle to the nerve roots was 2.0 ± 1.2 mm. No statistically significant differences between genders were found. **Conclusions**: An ultrasound-guided technique can be an accurate and safe technique to perform invasive procedures on the posterolateral part of the intervertebral lumbar discs.

## 1. Introduction

Low back pain is one of the most frequent musculoskeletal problems in the world [1]. About 55% of patients with low back pain also have pain that radiates to the lower limbs [2]. In addition, one of the most common causes of symptoms radiating to the lower limbs is intervertebral disc degeneration and herniated discs in the posterolateral area [3]. Approximately 40% of chronic low back pain patients have a discogenic origin [2]. Patients between 25 and 55 years old have approximately a 95% chance of herniated discs occurring either at L4-L5 or L5-S1 [4].

Minimally invasive techniques for the treatment of discogenic pain are receiving increasing attention due to their potential advantages [5]. These techniques include procedures such as nerve root blocks, epidural blocks, lumbar transforaminal epidural injections, lumbar discectomy, or intra-articular facet injections [6]. In recent years, there has been increasing interest in conservative invasive treatments focused on the intervertebral disc, especially through in-disc infiltration. Migliore et al. [2] recently reviewed disc injection treatments, noting their increasing popularity. They concluded that these techniques can be complex, expensive, and sometimes require inaccessible materials [2]. Indeed, among all the examined articles, most employ fluoroscopy for the procedure and others use computed tomography [2]. These techniques have some disadvantages, such as ionizing radiation for both patients and professionals, higher cost, and lower availability [5,7].

Advancements in ultrasound technology have increased their use for guided procedures, improving real-time tissue visualization and enhancing safety and efficacy [8]. Moreover, ultrasound devices are cost-effective, portable, and eliminate ionizing radiation associated with fluoroscopy or computed tomography. These advantages drive efforts to enhance the adoption of ultrasound guidance for lumbar spine procedures [6]. However, doubts about its validity still persist, as the ultrasound-guided technique for visualizing vertebral structures in the lumbar spine can pose challenges. This is primarily due to the three-dimensional complexity of their anatomy, coupled with the high acoustic impedance of vertebral references [5].

Most intervertebral disc injuries occur in the posterior and lateral zone of the disc, involving the anterior lumbar roots and the spinal cord [9]. Several articles point out that one of the most important aspects is the precision in reaching the affected target structure to achieve good results [2,10]. Traditionally, in medicine, disc infiltrations are typically performed directly into the nucleus pulposus, and precise needle placement into the exact hernia region is not deemed critical. In recent years, a novel technique called percutaneous electrolysis has been developed [11]. This technique involves applying a controlled galvanic current to the exact site of the lesion using an ultrasound-guided needle. Different studies have observed that this treatment induces a highly controlled local inflammation and promotes the recruitment of macrophages to eliminate damaged tissue [12,13,14]. This concept is particularly intriguing, as the literature suggests that spontaneous resorption of disc herniations occurs when local immune processes are activated at the site of the lesion, leading to macrophage recruitment [15,16,17]. Therefore, employing a precise technique in the herniation region using this method could be beneficial for hernia elimination. To the best of the authors’ knowledge, there are only two “case report” studies that evaluate the efficacy of using invasive ultrasound-guided techniques in lumbar intervertebral discs [18,19]. However, prior information on technique performance, including needle angulation, insertion depth to reach lumbar discs, and safety regarding needle proximity to lumbar spine structures, remains insufficient.

The present anatomical study aimed to analyze and describe the accuracy and safety of a new ultrasound-guided approach for targeting the posterolateral aspect of lumbar intervertebral discs in cadaveric specimens. Additionally, it sought to evaluate gender-based differences. We hypothesized that the ultrasound approach to reach this part of intervertebral lumbar discs in cadaveric specimens could be performed accurately and safely without gender differences. Furthermore, variables related to the technical procedure (insertion point, angulation, and depth of the needle) were analyzed, which may serve as guidelines to facilitate its replication.

## 2. Materials and Methods

### 2.1. Cadaveric Sample

A cross-anatomical study was designed with sixty (n = 60) intervertebral lumbar discs (twelve discs at each lumbar level L1-L2-L3-L4-L5) from twelve cryopreserved body donors to the anatomy laboratory of the Universitat Internacional de Catalunya. Inclusion criteria required that the bodies arrive at the laboratories within 24 h of death and be over 18 years of age. Exclusion criteria included the presence of abdominal or lumbar scars, need for autopsy, morbid obesity, or a history of infection or contagious pathologies. The bodies were preserved at −20 °C and acclimatized to room temperature 48 h before starting the research. This study was approved by the Local Ethics Committee (CBAS-2023-10). All donors signed a research body donation consent form in accordance with the country’s regulations.

### 2.2. Sample Size

A previous pilot study was conducted with twenty intervertebral lumbar discs to estimate the technique’s accuracy, achieving 75% accuracy in reaching the intervertebral lumbar discs. The previous literature suggests that the error level in optimal needle placement for these techniques ranges between 5.3% [20] and 15% [21].

The sample size was calculated with GRANMO V.7.12 assuming an infinite population, a technique precision of 0.75, and an error of 0.11. A sample size of sixty intervertebral lumbar discs was established.

### 2.3. Ultrasound-Guided Needling Approach

The ultrasound-guided needling approaches were performed by an experienced therapist with more than ten years of experience in ultrasound-guided invasive techniques. The approach aimed to place the tip of the needle in the posterolateral part of the different intervertebral lumbar discs. An ultrasound device (General Electric LOGIQ e R8) with a convex transducer (1–5 MHz) and beveled needles of 30 G X 7.5–12 cm were used.

**Figure 1:**
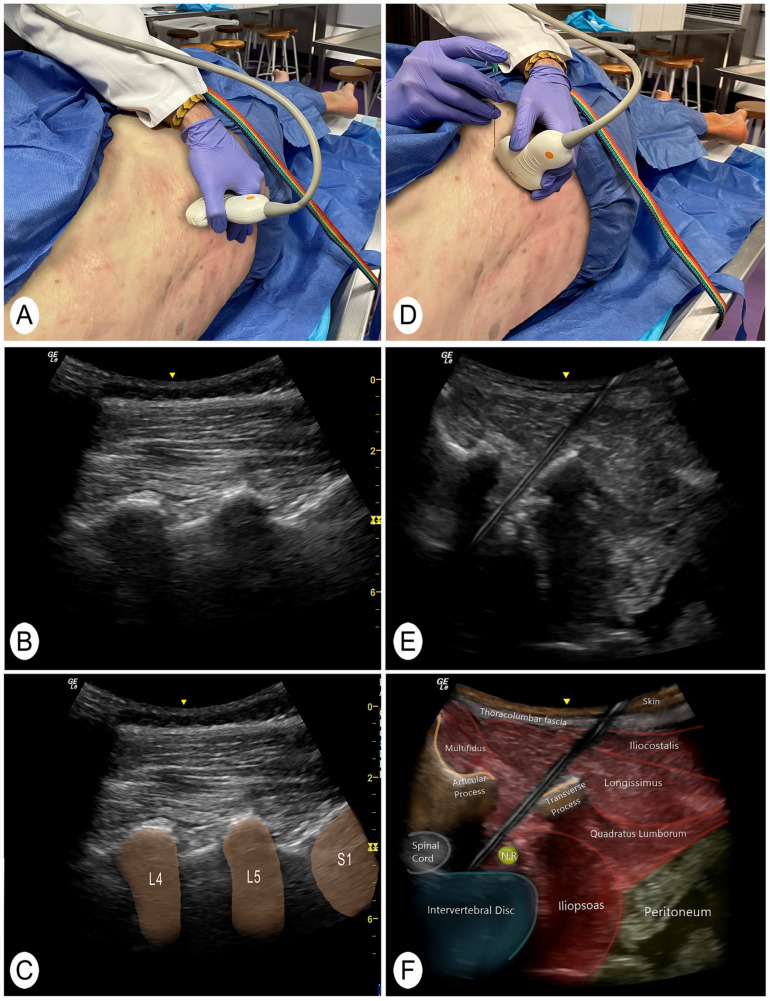
Posterior and lateral ultrasound of the invasive technique. Abbreviations: N.R., nerve root. L4, 4th lumbar vertebra; L5, 5th lumbar vertebra; S1, 1st sacral vertebra. (**A**) Position to locate the vertebral level with the transducer in a longitudinal slice of the facet joints. (**B**) Ultrasound image of the longitudinal section of the lumbar articular facets. (**C**) Ultrasound image of the longitudinal section of the lumbar articular facets with the vertebral segments identified. (**D**) Position of the transducer to perform the invasive technique in a posterolateral transverse section of the intervertebral disc. (**E**) Cross-sectional ultrasound image from a posterolateral view where the invasive technique of the intervertebral disc is performed. (**F**) Ultrasound cross-sectional identification of the structures.

**Figure 2:**
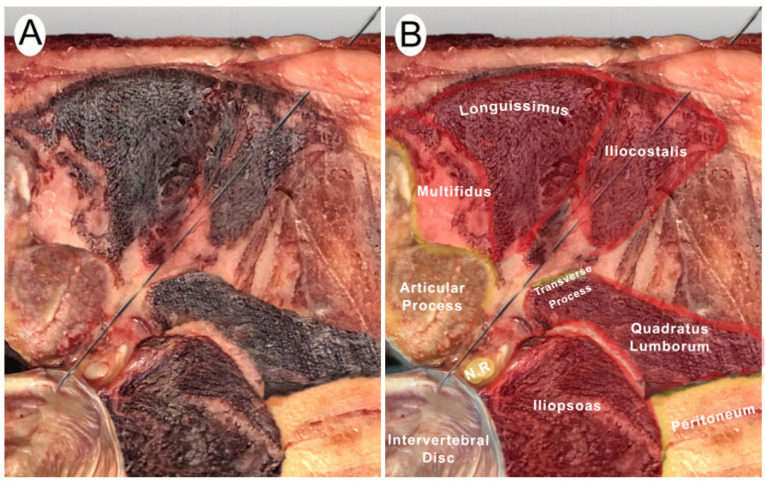
Posterior and lateral anatomic cross-section of the invasive technique. Abbreviations: N.R., nerve root; S.C., spinal cord. (**A**) Anatomic cross-section for the needling procedure. (**B**) Anatomic identification of the structures.

A posterior to anterior and lateral to medial approach was performed with the cadaver in lateral decubitus. To identify the target intervertebral segment, the sacrum was located manually. The transducer was then placed in a longitudinal plane of the spine to locate the facet joints (Figure 1A–C) [22]. With this image, the sacral bone and the intervertebral spaces could be clearly identified. This technique for identifying vertebral segments has been shown to be effective in the previous literature [22]. The target intervertebral space was then placed in the center of the screen and a cross-section was made with the ultrasound transducer. In this section, the spinous process, the lamina, the articular facet, and the anterior nerve root of the targeted segment were visualized [5]. The transducer was then moved slightly laterally until a clear image of the anterior root and intervertebral disc was obtained. In addition, an obliquity was generated with the lateral part of the transducer to avoid the transverse processes of the vertebrae. In the case of L5, the lateral end of the ultrasound transducer was moved slightly in a cranial direction to avoid the iliac bone. The needle was then introduced until it reached the posterolateral part of the intervertebral lumbar disc (Figure 1D–F and Figure 2A,B).

**Figure 3:**
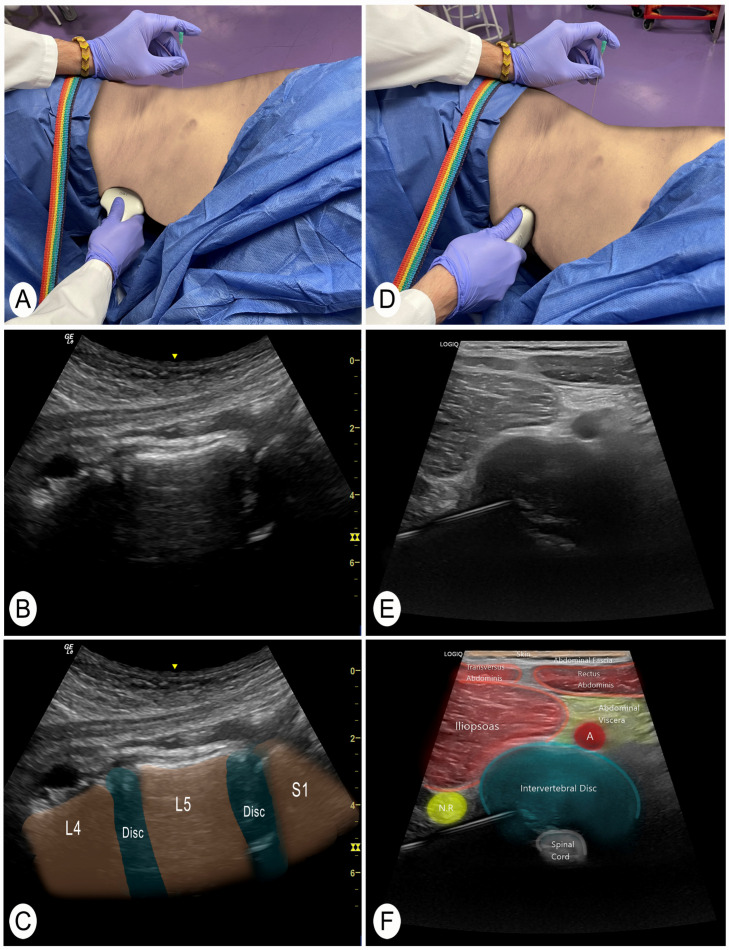
Anterior and slightly lateral view of the intervertebral disc. Abbreviations: N.R., nerve root; A, artery. L4, 4th lumbar vertebra; L5, 5th lumbar vertebra; S1, 1st sacral vertebra. (**A**) Position to locate the vertebral level with the transducer in a longitudinal slice of the vertebrae from the abdomen. (**B**) Ultrasound image of the longitudinal section of the lumbar levels from a ventral view. (**C**) Ultrasound image of the longitudinal section of the lumbar levels from a ventral view with the vertebral segments identified. (**D**) Position of the transducer in a cross-section of the intervertebral disc previously punctured. (**E**) Cross-sectional ultrasound image of the intervertebral disc from a ventral view where the previously inserted needle is located and adjusted. (**F**) Ultrasound cross-sectional identification of the structures.

The same procedure was performed at each lumbar segment. When performing this technique on live subjects, it is advisable to use the power Doppler function to identify vascular structures and not to pass through them. Once this check has been performed, the needle is placed in the center of the transducer (in-plane). During needle insertion, the tip of the needle must always be visualized until contacting the posterolateral part of the intervertebral disc. The nerve root should also be visualized to avoid perforating it. When the needle is slightly introduced into the intervertebral disc, a fibrous texture can be felt that confirms this structure. The ultrasound image was then saved for further measurements. The transducer is then removed and placed in the patient’s abdomen (the needle remains inserted, only the transducer moves) (Figure 3A). The objective is to visualize the needle from the patient’s abdomen. To do this, the target segment is located again, but now from the patient’s abdomen. The process starts with a longitudinal cut of the spine to identify the vertebral levels (Figure 3A–C). When the segment where the needle is located is identified, a cross-section of that intervertebral disc is performed to visualize the needle in plane (Figure 3D–F). Finally, final adjustments are made to the needle to bring it to the target disc area. A second ultrasound image is then saved for further measurements.

### 2.4. Anatomical Procedure

Once all the needles had been guided by ultrasound, a dissection was performed to check the final position of the needle tips. The dissection was performed from the anterior abdominal side.

Nearby viscera were checked to ensure that the punctures had not reached any of them. Then, the tissue was cleaned until the lumbar intervertebral discs could be observed.

Ultrasound punctures were also checked to ensure they matched the target segment. This verification was possible because the dissection was performed from the abdominal side of the donor. This situation allowed the examiner, in case of doubt, to reposition the transducer on the posterior side and see the needle and the vertebral disc dissection from the ventral side in real time.

### 2.5. Outcome Variables

Age, gender, and the abdominal perimeter of the specimens were recorded as demographic variables. The accuracy of the described technique to reach the posterolateral part of the lumbar intervertebral discs was recorded as the main variable. These data were obtained by analyzing the ultrasound images of the vertebral disc (Figure 2). The intervertebral disc was divided into four parts and the region in which the needle was located was evaluated.

- Reach the intervertebral disc (yes/no).
- Region of the intervertebral disc (anterolateral, posterolateral, anteromedial, or posteromedial).

In addition, the following variables were recorded [5]:

- The length of the needle introduced into the body (mm).
- The distance from the vertebral spinous process to the needle’s point of entry (mm) with ultrasound measurement.
- The angulation of the needle with the skin surface (in degrees) with photographic measurement.
- Unwanted structures crossed with anatomical dissection check-up.
- Distance of the needle to the main nerve roots (mm) with ultrasound measurement.

A reliability study was conducted with ten photos to verify the accuracy of the photographic measurement. The pilot study showed excellent reliability (ICC 0.98, 95% 0.97–0.99).

### 2.6. Statistical Analysis

Statistical analysis was performed with the SPSS statistical package (version 25.0). Means and standard deviations (SD) were calculated for each evaluated distances. A descriptive percentage analysis was performed to determine the number of needles that reached the posterolateral part of the discs. A preliminary gender analysis was conducted by applying the ANOVA test for the comparison between males and females.

## 3. Results

The ultrasound-guided needle technique was performed on sixty intervertebral lumbar discs (twelve discs at each lumbar level L1-L2-L3-L4-L5). In this sample, 50% were men and 50% were women, with a mean age of 74 ± 10 years and an abdominal perimeter of 39 ± 9 cm.

Overall (n = 60 lumbar intervertebral discs), the objective of reaching the posterolateral zone was achieved in 93.3% of the cases. In one approach (1.7%) the needle went to the slightly anterior part of the disc at L1. In three discs the technique could not be performed, due to one case of the presence of lumbosacral transitional vertebra (1.7%) at L5 and two cases in which there was no vertebral disc (3.3%) at L4 and L5.

The mean of the inserted length of the needle into the skin was 79 ± 15 mm, with a distance of 77 ± 19 mm on average to the spinous processes and an angle of 129.0 ± 20.2. The average (mean) distance between the needle and the nerves was 2.0 ± 1.2 mm. Only one L5 segment root was punctured. The distances to the nerve root at the L5 segment were the smallest despite no statistically significant differences with the distance to the nerve roots of other segments. The descriptive values of the puncture of each vertebral disc (L1-L2-L3-L4-L5) can be found in Table 1.

In the comparative analysis between genders, no statistically significant differences were found in any of the variables studied at either the segmental or global level.

## 4. Discussion

The current anatomical study aimed to analyze and describe the accuracy and safety of an ultrasound-guided approach to target the posterolateral part of the different intervertebral lumbar discs in cadaveric specimens and to assess whether differences exist between genders. The results indicated that this approach is accurate in 93.3% of the cases and that there were no differences between genders when performing the technique. The present study explains the steps to visualize the disc and to perform the invasive technique focused on the posterolateral part, providing guidance on needle insertion point, angulation, and depth, which can be utilized as guidelines to facilitate its subsequent replication. However, the proximity to the different anterior roots (2.0 ± 1.2 mm) should be considered. These indications could be interesting for various invasive treatments of lumbar intervertebral disc pathology such as injection with oxygen–ozone mixture, saline solution, steroids (methylprednisolone, acetate of prednisolone, hydrocortisone, betamethasone), and local anesthetic (bupivacaine, lidocaine) [2]. This approach is especially relevant for interventions where the precision of the effect is crucial [12,13,14]. An example of such techniques is the percutaneous electrolysis previously explained in the Section 1. Although this study did not involve the use of medications, our findings lay the groundwork for future clinical applications, where specific drugs and dosages will need to be determined for precise and safe interventions.

In the present study, the aim was not achieved in 6.7% of the cases. This was due to the presence of a lumbosacral transitional vertebra in one L5 segment and two cases in which there was no vertebral disc at L4 and L5. Ruangchainikom et al. [23] provide data on intervertebral disc degeneration measured with MRI. They explain that degeneration increases with age and that the L5 (61.5%) and L4 (55.0%) discs have a much higher prevalence of degeneration than the others [23]. This degeneration can lead to the complete disappearance of the disc, especially in the elderly population [23]. Degeneration may be combined with anatomical abnormalities, such as the presence of a lumbosacral transitional vertebra. Shaikh et al. [24] performed a study with radiography and MRI with 504 patients. They observed that a lumbosacral transitional vertebra was present in 15% of patients [24]. Disc degeneration and anatomical abnormalities often occur together, and it is estimated that these variations are present in 4% to 30% of the population [25]. Our sample was composed of cadavers, which results in an older sample population and could justify these findings (6.7%) in the L4 and L5 segments. In these cases, ultrasound visualization of the lumbar intervertebral disc is not possible and would require intervention with fluoroscopy or computed tomography [5].

Most studies that perform invasive techniques on the disc aim to reach the posterolateral area through computed tomography, magnetic resonance, or fluoroscopy [2]. However, the present study provides not only a posterolateral invasive approach, but also a more complete view of the intervertebral disc that allows more precise corrections to be made in real time through ultrasound. To the best of the authors’ knowledge, there are only two “case report” studies that evaluate the efficacy of using invasive ultrasound-guided techniques in lumbar intervertebral discs [18,19]. These studies with low sample sizes do not provide descriptive data on safety such as distances to relevant structures during injection or the accuracy of the posterolateral location of the needle.

Ultrasound-guided needle placement is an acquired skill with a relatively long operator-dependent “learning curve” [5,26]. According to a recent review on invasive spinal techniques, all studies performed are expert-driven due to the complexity of the approaches [5,27], which underscores the importance of expertise when performing this type of technique.

The use of ultrasound for guiding invasive procedures is clinically significant due to its non-ionizing nature, cost-effectiveness, high availability, and portability. Additionally, ultrasound provides clear and efficient visualization of soft tissues and blood vessels (using Doppler ultrasound), offering a valuable advantage in specific situations. While complications have been reported with fluoroscopy and computed tomography during lumbar nerve root interventions, techniques like digital subtraction angiography are employed to mitigate such issues [28]. Consequently, ultrasound guidance emerges as a viable method to prevent vascular injuries in intervertebral lumbar disc procedures. However, the precision of ultrasound-guided interventions in the lumbar spine may be influenced by technical limitations, the depth of nerve roots, and individual patient characteristics [6]. These findings suggest that alternative guidance techniques may be required to ensure accurate targeting of the intervertebral lumbar disc when ultrasound visualization is not optimal.

This study presents some limitations, which are described below. In clinical practice, the patient can “tuck in the navel” to reduce abdominal circumference and improve anterior visualization of the vertebral disc (which was not possible in the specimens). As this was a cadaveric study, Doppler ultrasound, which could provide information on blood vessels to avoid along the needle trajectory, could not be utilized. Similarly, the sample did not exhibit physiological movements (voluntary and involuntary somatic and visceral) that could hinder the technical execution. There is a relationship between the presence of a lumbosacral transitional vertebra, intervertebral disc degeneration, and low back pain. However, since it is a cadaveric sample and access to the patient’s entire clinical history is not available, it is not known whether the subjects in the study had low back pain.

## 5. Conclusions

The ultrasound-guided intervention described could be a precise and safe technique to perform invasive interventions in the posterolateral region of the different lumbar intervertebral discs. No statistically significant difference was found between genders. The distance to the nerve roots should be carefully considered, and good visualization of these structures is essential, given the small distance to the needle.

## Figures and Tables

**Table 1:** Descriptive values.

	L1 (n = 12)	L2 (n = 12)	L3 (n = 12)	L4 (n = 12)	L5 (n = 12)	Global (n = 60)
Length of the needle introduced (mm)	75 ± 9	73 ± 15	81 ± 12	86 ± 16	81 ± 18	79 ± 15
Angulation of the needle (°)	134.9 ± 23.8	137.7 ± 14.3	131.0 ± 20.4	124.5 ± 18.8	116.7 ± 15.3	129.0 ± 20.2
Reach the posterolateral part of the discs	91.7%	100%	100%	91.7%	83.3%	93.3%
Distance from the vertebral spine to the needle (mm)	80 ± 15	78 ± 15	83 ± 22	77 ± 23	67 ± 20	77 ± 19
Distance of the needle to the main nerve roots (mm)	2.1 ± 0.6	2.2 ± 0.8	2.3 ± 2.0	1.8 ± 1.0	1.6 ± 0.7	2.0 ± 1.2
Unwanted structures crossed	-	-	-	-	1 nerve root	1 nerve root

## Data Availability

Data are available upon request to the corresponding author.
